# EFFECT OF INITIATING ASPIRIN ON CANCER EVENTS IN THE HEALTHY ELDERLY

**DOI:** 10.1093/geroni/igz038.2358

**Published:** 2019-11-08

**Authors:** Andrew T Chan, Peter Gibbs, Jessica E Lockery, Galina Polekhina, Suzanne G Orchard, Leslie Ford, Asad Umar, john J McNeil

**Affiliations:** 1 Clinical and Translational Epidemiology Unit, Massachusetts General Hospital, Boston, Massachusetts, United States; 2 The Walter & Eliza Hall Institute of Medical Research, Parkville VIC, Victoria, Australia; 3 Department of Epidemiology & Preventive Medicine, Monash University, Clayton VIC, Victoria, Australia; 4 Division of Cancer Prevention, National Cancer Institute, Bethesda, Maryland, United States

## Abstract

In ASPREE, we previously reported a surprising increase in cancer-related deaths associated with initiating aspirin. We now report primary incident cancer events. Aspirin was not associated with risk of incident cancer (HR=1.04, 95% CI 0.95-1.14), including non-metastatic cancer (HR=0.99, 95% CI, 0.89-1.11) and colorectal cancer (HR=1.02; 95% CI, 0.81-1.30). However, risk of incident metastatic cancer was elevated with aspirin (HR=1.18; 95% CI,0.96-1.46), although this could be attributable to chance. In ASPREE, the increase in cancer deaths associated with initiation of aspirin was not accompanied by a significant increase in overall incident cancer after 4.7 years. However, there did appear to be an increase in the incidence of advanced cancer in the aspirin arm. These data support the possibility that aspirin may adversely affect short-term outcomes among elderly participants with undiagnosed cancers (e.g. prevalent tumors at enrollment or early incident tumors) and/or may have differential effects according to age.

